# Assessing the Importance of Psychosocial Factors Associated With Sustainable Organizational Development During COVID-19

**DOI:** 10.3389/fpsyg.2021.647435

**Published:** 2021-03-11

**Authors:** Florin Dragan, Chaoping Luo, Larisa Ivascu, Majid Ali

**Affiliations:** ^1^Department of Automation and Applied Informatics, Faculty of Automation and Computers, Politehnica University of Timisoara, Timisoara, Romania; ^2^College of Economics and Management, Southwest University, Chongqing, China; ^3^Department of Management, Faculty of Management in Production and Transportation, Politehnica University of Timisoara, Timisoara, Romania; ^4^Research Center in Engineering and Management, Timisoara, Romania; ^5^Hailey College of Commerce, University of the Punjab, Lahore, Pakistan

**Keywords:** sustainability, innovation, shareholder, theory of planned behavior, motivation, attitude, behavior, COVID-19

## Abstract

Involvement in sustainable development is a voluntary activity. Organizations apply the principles of sustainable development only when they identify several benefits. These benefits are identified, especially with the financial ones. The involvement of organizations in sustainable organizations has different intensity levels. These intensity levels are influenced by psychosocial factors (PF), attitudes toward organizational risks, and organizational and urban policies. The present paper identifies the key psychological factors involved in applying organizational sustainability principles within organizations. For this research, five groups were created for in-depth interviews with key people from Romania’s innovative companies. To identify the importance of the framework analysis factors, the Delphi method was used, in which 20 experts from different fields of activity were involved. Following the rounds involved in the Delphi method, the ranking of PF on four levels of importance was accepted, based on planned behavior and reasoned action theory. These levels were correlated with the intensity levels of involvement in sustainable development. The entire market study was conducted during COVID-19, which significantly impacted specific directions. As a result, it could be observed that motivation, learning attitude, behavior, and intention to take precedence are essential in the organizational sustainability approach.

## Introduction

Sustainability means that a company can secure its long-term resources, anticipate its future needs, and use resources efficiently and fairly in organizational activities. This is a reference definition, developed in 1987 by Norwegian Prime Minister Gro Harlem Brundtland in the report entitled “Our Common Future,” prepared by the United Nations World Commission on Environment and Development. Sustainability does not only mean ecology, it also means achieving a balance between the three responsibilities: economic, social, and environmental. These responsibilities are embedded in most existing definitions in the literature, where there are several concerns for social equity and economic development (Horn et al., 2012; Noone et al., 2018; Pislaru et al., 2019).

Involvement in sustainable development differs from one company to another. Some of the companies are heavily involved, with several benefits. Other companies get involved for specific criteria they want to achieve (requiring employees to report their sustainability or have an adequate environmental footprint). It can be seen that in the manufacturing industry (Labuschagne et al., 2005; Jayal et al., 2010; Hesselbach and Herrmann, 2011; Mani et al., 2014; Ford and Despeisse, 2016; Kayikci, 2018), the implications are numerous. Over the years, this industry has shown an increasing evolution from involvement. The construction sector (Naik, 2008; Abidin, 2009; Marhani et al., 2012; Yılmaz and Bakı, 2015; Molavi and Barral, 2016; Kamali and Hewage, 2017) has many implications. Within this sector, the implications refer to buildings’ energy efficiency and the use of adequate construction materials. The electricity and heat sector, gas, hot water, and air conditioning are making considerable progress in sustainability principles (Oparaocha and Dutta, 2011; Sinclair et al., 2015; Dincer and Acar, 2017; Kveselis et al., 2017). Here, steps are being taken to reduce greenhouse gasses generated in the atmosphere. They began to use innovative technologies that consider consumer-dependent factors (Blevis, 2007; Barquet et al., 2016; Lal, 2016; Arfini et al., 2019). Studies conducted in the machine-building industry have highlighted the need to investigate psychosocial factors (PF) to increase the level of involvement in OS. Factors are mentioned as motivation and attitude (Cioca et al., 2015). The transport sector is also making progress due to new technologies used in vehicle manufacturing (Richardson, 2005; Steg and Gifford, 2005; Anderton, 2010; Yigitcanlar and Dur, 2010). Pollution norms contribute to reducing the level of greenhouse gasses and fuel efficiency. In this way, combined with driver-dependent factors, efficient use of resources in the field of transport (Steg and Gifford, 2005; Anderton, 2010; Cruijssen, 2020). It can be seen that the implications of organizations in different fields are numerous (Al-Mulali and Ozturk, 2015; Apergis and Ozturk, 2015; Farhani and Ozturk, 2015; Ozturk, 2017). The involvement of organizations in this voluntary approach depends on several factors. These factors include psychological factors, social factors, economic factors, technological factors, and other factors. Psychological factors are considered some of the most important, especially when it comes to the entry barrier. Among the social factors that contribute to the success of adopting approaches for sustainability are perception, motivation, learning, attitudes, beliefs, self-efficacy, culture, and others (Abdullah et al., 2017, 2018; Sarfraz et al., 2018a,b). Being a voluntary approach, the social factors’ approach presents a necessity in successfully implementing the principles of sustainability (Bocken et al., 2014; Eccles et al., 2014; Adloff, 2019).

The benefits involved in the organizational sustainability are diverse and multiple. First of all, there can be an improvement in financial results. These benefits are most attractive to organizations. Also, the following can be achieved: efficiency of processes and activities, increasing the level of innovation, reducing the carbon footprint and water, waste management, growing capacity of globalization, improving employee skills, strengthening work teams, aligning with global requirements, attracting new business partners, identifying new organizational opportunities, fulfilling an international vision, improving the image, and others (Apergis and Ozturk, 2015; Ivascu, 2020).

Several studies emphasize the need to identify PF. Identifying these factors can contribute to an accentuation of their treatment to intensify the involvement of organizations in organizational sustainability (Ivascu, 2020). There are several studies on organizational sustainability in Romania, but studies on PF are very few. These existing studies target the industry with a few limitations. Therefore, we have identified a need for a national study to help organizations identify and improve their PF for OS. In this variant, the organizational implications in sustainability can be intensified, and the organizations can increase their level of innovation (Cioca et al., 2015; Ivascu, 2020).

This research aims to identify PF at the level of organizations in Romania and improve the level of intensity of organizations for involvement in organizational sustainability. These intensity levels are influenced by PF, attitudes toward organizational risks, and organizational and urban policies. This study can be customized for specific activity fields or branches of activity. This study can be customized for specific activity fields or branches of activity and represents a pillar in intensifying the levels of the organization. Based on a transdisciplinary approach, the applied methodology allows the generation of a nuanced interpretation of problematic implications. It addresses the gaps in psychology that apply to sustainable organizational development. It enhances organizations’ innovation. In-depth interviews were analyzed with framework analyses. The paper begins with the presentation of the essential research in sustainability. It continues with the importance of innovation and involvement in OS, matrix presentation of factors identified in interviews, and developing the logic of intensity levels developed due to applying the Delphi method. Finally, the main elements found are presented. The conclusions refer to the practical and academic contributions within this organizational sustainability field.

## Literature Review

Involvement in sustainability involves innovative approaches that develop several benefits for the organization. One cannot discuss sustainability without innovation and vice versa. It is a two-way relationship that supports PF for organizational involvement. Given this relationship, the chapter is structured on the following directions: the presentation of organizational sustainability, the importance of innovation for sustainability, and organizational theories applicable in companies (Linnenluecke and Griffiths, 2010; Liu et al., 2015).

### Sustainable Organization

Sustainability and sustainable development as concepts have gone through different development stages since their introduction. The historical development of the concept has taken place at various conferences, within organizations and institutions, which are currently concerned with implementing the principles, goals, and objectives of sustainable development. The concept of sustainable development has known various criticisms and interpretations over time, being accepted in various activity fields (Sadiq et al., 2020). In its development, the concept has adapted to a complex global environment’s contemporary requirements. Still, the basic principles and objectives, as well as the problems of their implementation, have remained almost unchanged. However, some goals have been updated, and new goals have been set since 2015. These goals are united in the 2030 Agenda, which outlines the challenges that humanity must meet for sustainable development, but also to survive on the planet (Choong and McKay, 2014; Erdmann Hilty et al., 2004; Ma et al., 2014; Honda, 2020).

In the 18th century, economic theorists, such as Adam Smith, pointed out organizational development problems. In the 19th century, Karl Marx and the classical economists Malthus, Ricardo, and Mill argued for certain sustainable development elements. In contrast, economic theory Neoclassical stressed the importance of pure air, water, and renewable resources (fossil fuels, ores), as well as the need for government intervention in extraordinary cases. Previous periods have dominated economic doctrine dominance, emphasizing human capital as a natural resource pilot (Smith et al., 2010; Tainter, 2011; Strathern and Stewart, 2018).

The term “sustainable development” was initially introduced in forestry and included afforestation measures for interconnected forests, which should not undermine forests’ biological renewal. This term was first mentioned in the International Union for Conservation of Nature (IUCN) Strategy for Nature and Natural Resources (IUCN), published [International Union for Conservation of Nature (IUCN) (2020)].

Sustainable development actions target 17 objectives (SDGs) and 169 targets (Lim et al., 2016; Pradhan et al., 2017; Schroeder et al., 2019). The companies partially aim at these objectives included in the 2030 Agenda. By evaluating the 17 objectives and the PF that influence the organizational implications, the authors perform an analysis presented in Table 1.

**TABLE 1:** Evaluation of the 17 SDGs and psychosocial factors.

**Goal**	**Short description**	**Implications of psychosocial factors**
SDG 1	Eradicate poverty in all its forms	Perception (P), motivation (M), learning (L), perceived behavior control (CPB), attitude (A), belief (B), norms applicable to the field (NSAF), and behavioral intent (BI)
SDG 2	Food safety and sustainable agriculture	subjective norms (SN), the perceived power of the approach (PPA), behavioral intention (BI), norms especially applicable to the field (NSAF), perceived power of approach (PPA), and subjective norms (SN)
SDG 3	Healthy life at all ages	Perception (P), motivation (M), learning (L), perceived behavior control (CPB), attitude (A), belief (B), norms applicable to the field (NSAF), and behavioral intent (BI)
SDG 4	Lifelong learning	Perception (P), motivation (M), learning (L), perceived behavior control (CPB), attitude (A), belief (B), norms applicable to the field (NSAF), and behavioral intent (BI)
SDG 5	Equality between women and men	Subjective norms (SN), the perceived power of the approach (PPA), norms applicable to the field (NSAF), and behavioral intent (BI)
SDG 6	Sustainable water management and sanitation for all	Perception (P), motivation (M), learning (L), norms applicable to the field (NSAF), and behavioral intent (BI)
SDG 7	Affordable prices and sustainable resources	Subjective norms (SN), the perceived power of the approach (PPA), attitude (A), belief (B), and norms applicable to the field (NSAF)
SDG 8	Promoting economic growth, productive and decent work	Norms applicable to the field (NSAF), behavioral intent (BI), attitude (A), and belief (B)
SDG 9	Promoting industrialization and stimulating innovation	Perception (P), motivation (M), learning (L), perceived behavior control (CPB), attitude (A), belief (B), norms applicable to the field (NSAF), and behavioral intent (BI)
SDG 10	Reducing inequalities between countries	norms applicable to the field (NSAF) and behavioral intent (BI)
SDG 11	Developing secure living environments	Attitude (A), belief (B), and norms applicable to the field (NSAF)
SDG 12	Sustainable consumption and efficient production	Perception (P), motivation (M), learning (L), and behavioral intent (BI)
SDG 13	Urgent action to combat climate change	Subjective norms (SN), the perceived power of the approach (PPA), perception (P), motivation (M), and learning (L)
SDG 14	Sustainable use of marine resources	Perception (P), motivation (M), learning (L), and behavioral intent (BI)
SDG 15	Restoration of terrestrial ecosystems	Subjective norms (SN), the perceived power of the approach (PPA), perception (P), motivation (M), learning (L), norms applicable to the field (NSAF), and behavioral intent (BI)
SDG 16	Responsibility of society and equity of institutional levels	Behavioral intent (BI), subjective norms (SN), the perceived power of the approach (PPA), perception (P), motivation (M), and learning (L)
SDG 17	Partnerships for the goals	Subjective norms (SN), the perceived power of the approach (PPA), norms applicable to the field (NSAF), and behavioral intent (BI)

### The Importance of Innovation for Organizational Sustainability

Innovation has a vital role in the current dynamics of the business environment. Innovative organizations aim at a series of objectives and targets of sustainable development. At the level of Romania, innovative enterprises’ situation is presented in Table 2 (National Institute of Statistics, 2020). It can be seen that the manufacturing industry and services present innovative enterprises for the analyzed period.

**TABLE 2:** The situation of innovative enterprises in Romania (National Institute of Statistics, 2020).

**Activities**	**Year**				
	**2015**	**2016**	**2017**	**2018**	**2019**
	
	**Number**	**Number**	**Number**	**Number**	**Number**
Total	8,116	5,968	3,645	2,925	4,198
Industry	4,439	3,415	1,843	1,493	2,298
Extractive industry	61	45	21	2	13
Manufacturing industry	4,143	3,200	1,754	1,455	2,176
Production and supply of electricity and heat, gas, hot water, and air conditioning	71	58	26	11	37
Water distribution, sanitation, waste management, decontamination activities	164	112	42	25	72
Services	3,677	2,553	1,802	1,432	1,900

From the perspective of innovators in the analyzed period 2015–2019, in Romania, the situation is presented in Table 3. Product and process innovators are among the first to innovate. At the same time, innovative business processes innovate and are in a favorable position. The number of innovators has increased to 4,198 in 2020 (National Institute of Statistics, 2020).

**TABLE 3:** Types of innovators in Romania (National Institute of Statistics, 2020).

**Types of innovators**	**Year**						
	**2015**	**2016**	**2017**	**2018**	**2019**	**2020**	**2020**
	
	**Number**	**Number**	**Number**	**Number**	**Number**	**Number**	**Procent**
Total	3,983	3,763	1,806	1,840	1,556	4,198	100
Product innovators only	582	635	351	313	430	486	11.58
Process innovators only	413	955	706	511	478	600	14.30
Only business process innovators	–	–	–	–	–	1,281	30.51
Product and process innovators	2,968	2,041	634	705	518	1,250	29.78
Only product innovators and business processes	–	–	–	–	–	507	12.08
Innovators with unfinished and or abandoned activities	20	132	115	311	130	68	1.62
Enterprises without innovation, but with abandoned/suspended or ongoing innovation activities	–	–	–	–	–	6	0.14

From the perspective of size, the situation is presented in Table 4. It can be seen that small enterprises register the most crucial level of innovation. Medium-sized enterprises follow them. The industry has an essential impact on innovation’s perspective (National Institute of Statistics, 2020).

**TABLE 4:** The situation of innovative enterprises by size classes (National Institute of Statistics, 2020).

**Size classes and economic activities**	**Year**					
	**2015**	**2016**	**2017**	**2018**	**2019**	**2015**
	
	**Number**	**Number**	**Number**	**Number**	**Number**	**Number**
Total	3,983	3,763	1,806	1,840	1,556	4,198
Small	2,137	2,386	1,034	1,258	1,030	3,022
Medium sized	1,183	936	506	371	373	825
Big sized	663	441	266	211	153	351
Industry	2,907	2,381	1,141	937	858	2,298
Services	1,076	1,382	665	903	698	1,900

Table 5 presents the share of enterprises with organizational innovation in the main activity fields. The manufacturing industry holds a vital percentage, followed by the production and supply of electricity and heat, gas, hot water, air conditioning, and services. The whole situation is presented below (National Institute of Statistics, 2020).

**TABLE 5:** Percentage of enterprises with organizational innovation by activities (National Institute of Statistics, 2020).

**Activities**	**Year**				
	**2015**	**2016**	**2017**	**2018**	**2019**
	
	**Percent**	**Percent**	**Percent**	**Percent**	**Percent**
Total	20.8	18.4	14.1	6.7	5.8
Industry	20.5	17	15.3	6.7	6.2
Extractive industry	18.5	12.7	10	4.9	0.6
Manufacturing industry	20.5	16.9	15.6	6.9	6.7
Production and supply of electricity and heat, gas, hot water, and air conditioning	23.8	32.1	20.9	8.3	2.4
Water distribution, sanitation, waste management, and decontamination activities	20.4	16.9	11.3	3.2	1.2
Services	21.2	20.1	12.8	6.7	5.3

### Organizational Theories Applicable to the Present Research

The theory of Reasoned Action (TRA or ToRA) was developed by Martin Fishbein and Icek Ajzen, an improvement in information integration theory (Fishbein and Ajzen, 1975, 2010; Hill et al., 1977), a cognitive theory that presents a conceptual framework for understanding human behavior in different situations. This theory helps explain individuals’ behavior and involvement in various activities or decision-making situations. Behavioral intention (BI) is created or caused by two factors: attitudes (what is expected to be done and motivation) and subjective norms (SN) (strengthening the normative faith and persuasive objectives) (Madden et al., 1992; Lada et al., 2009; Trafimow, 2009; Rossmann, 2011; Hale et al., 2002). It is an extension of TRA, and the main component of this model is behavioral intent. Individual attitudes and subjective risk assessment influence these BIs. TPB consists of six constructions: attitudes, BI, SN, social norms, perceived power, and perceived behavioral control (Ajzen, 1985, 1991, 2012; Rizzo and Columna, 2020).

## Methodology

The research methodology is qualitative and transdisciplinary based on the experts’ empirical experience in sustainability. Below are the details of each step applied in the research presented. The entire research was carried out during (and has faced the impact of) the COVID-19 period, March–November 2020, when most of the organizational activities were carried out through telework. The companies involved in the present research are in categories A–S (presented in Appendix 1). The methods selected for this research have several advantages based on research. During this period of applying this study’s methods, a large part of the subjects involved in the study worked in telework. For each method used, the advantages are presented.

### In-Depth Interviews

This research method is a qualitative one and involves direct contact with each participant. In-depth interviews are used to collect data because they have several advantages, especially during the COVID-19 period. These advantages include the following: sensitive topics can record complex and clear answers, subjects will feel comfortable and answer all questions, key topics can be researched and can be returned to at any time, it is a superior quality for sampling, the number of respondents does not have to be very large to obtain remarkable and relevant results, there is no possibility of pressure from participants, interviewers can monitor the tone and words used by participants, and the degree of focus on the topics is higher (Guest et al., 2006; Longhurst, 2009; Campbell et al., 2013; Adams and Cox, 2016).

To conclude this initial research, five working groups were set up. In the five working groups, 150 interviewees from Romanian organizations apply sustainability principles in their operations. An expert in organizational sustainability interviewed each working group. This helped to avoid the loss of essential data. Among the advantages of this method are the following: collecting a large amount of data, the information obtained is qualified and conclusive, and coordinating the interviews in the intended direction. For in-depth interviews, 10 common questions and five different questions were established for each working group. The structure of these questions is presented in Table 6. The five different questions were established by the experts used in the application of the Delphi method. These questions were developed according to the companies’ particularities of activity that constitute each working group.

**TABLE 6:** The structure of in-depth interviews.

**Workgroup**	**Questions used**
Groups 1, 2, 3, 4, and 5 (common questions)	General questions regarding SO (implications on the Economic, Social, and Environmental Dimensions). Examples: When was the last sustainability report? What are the SDGs targeted by the organization?
Group 1	Fundamental concepts in innovation Example: What are the latest technologies implemented?
Group 2	Fundamental concepts related to behavioral theories (TPB and TRA) Example: How important is the person’s temperament in charge of OS?
Group 3	Questions regarding product innovation by industry categories in the fields of activity A–S Examples: Do you appreciate the level of innovation in the industry? What are the national factors that contribute to increasing the industry’s level of innovation?
Group 4	Questions regarding process innovation in the fields of activity A–S Examples: Do you appreciate the level of innovation in the field? Characterize the field with a sentence.
Group 5	Questions regarding the innovation of business processes in the fields of activity A–S Example: Define the organization’s business processes in terms of innovation.

### Delphi Method

The Delphi method involves a structured communication method that applies to a group of experts through the synchronous communication method. These experts evaluate the reports generated in several rounds of decisions until all experts agree with the variant generated. The method generates a final report of the decisions accepted by all experts. The Delphi method has several advantages, given in the present study. These advantages include the following: participants can be found anywhere in the world, a quick consensus of those discussed is obtained, it covers a wide range of expertise, avoids long interpretations and discussions, the predicted questions are specific and have predetermined answers, and you get a final report agreed by all experts during the study (Linstone and Turoff et al., 1976; Gordon and Pease, 2006; Botterill and Platenkamp, 2014; Chalmers and Armour, 2019; Humphrey-Murto et al., 2020). Find involved experts who consider that the final report is an adequate one and contains vital information for the targeted field. In the present research, 20 experts from the critical sustainability and innovation fields were involved. The expertise and their fields of action are presented in Table 7. The division of experts into groups was made according to the enterprises’ size involved in innovation.

**TABLE 7:** The structure of the experts involved in the application of the Delphi method.

**Expert typology**	**Field of activity and action**
Expert group 1	Experts with knowledge in innovation and sustainability in the industry Fields of activity: automotive, textiles, metallurgy, electrical appliances, furniture, waste recovery, etc.
Expert group 2	Experts with knowledge in innovation and sustainability from medium-sized enterprises Field of activity: textiles, furniture, commerce, education, transport, and others.
Expert group 3	Experts with knowledge of innovation and sustainability in small businesses Field of activity: textiles, furniture, commerce, small production, and others.
Expert group 4	Experts with knowledge in innovation and sustainability from large enterprises Field of activity: automotive, textiles, metallurgy, waste management, transport, and others.
Expert group 5	Experts with knowledge in innovation and sustainability in services Field of activity: distribution, consulting, event organization, provision of information technology services, programming, and others.

**FIGURE 1:**
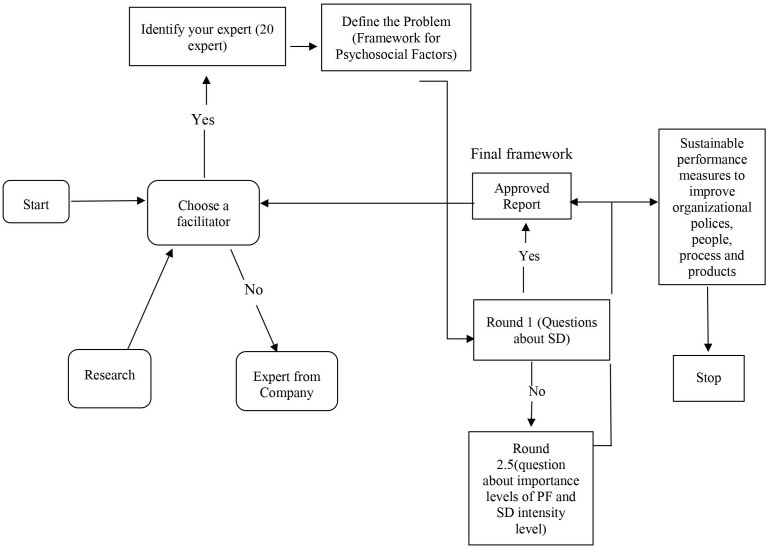
Application of the Delphi method in the present research.

The scheme of applying the Delphi method is presented in Figure 1. It can be seen in the proposed logical scheme that five rounds of in-depth discussions were held with experts in the field of SD and innovation to assess the levels of importance of PF identified and to achieve hierarchy and their correlation.

### Market Research and the Investigated Sample

The market research was conducted during COVID-19, March–November 2020. Given this period’s specifics, the study was conducted by the asynchronous method using online video conferencing platforms.

The investigated sample consisted of 150 representatives of companies with solid knowledge of innovation and sustainable development. The sampling was randomly applied to a group of 500 possible participants (organizations identified participants). The companies’ fields of activity are in the fields of A–S (presented in Appendix 1). The five working groups were formed by random sampling, presented in Table 8. In each group, 30 individuals were involved with solid knowledge in organizational sustainability. The five groups’ structure emphasizes that Romania’s essential fields of activity have been covered. The working groups’ structure was established based on the types of innovators evaluated for 2015–2019 and following the fields of activity presented in Appendix 1. These classifications are provided by the Romanian legislation, called nomenclature of activities. The working groups were correlated with the associated questions’ structure because for each working group, five personalized questions were used. Each group presents the percentages of the five most essential activity fields present.

**TABLE 8:** Working group structure.

**Working groups**	**Field of activity**
Group 1	Product innovators only in the fields of activity A–S (25% C, 15% D, 10% E, 10 % J, 8% S, and others)
Group 2	Process innovators only in the fields of activity A–S (35% C, 30% F, 25% E, 10% J, 10% E, and others)
Group 3	Innovators only of business processes in the fields of activity A–S (30% T, 10% L, 10% N, 10% P, 10% P, and others)
Group 4	Product and process innovators in the fields of activity A–S (25% C, 20% E, 10% D, 10% B, 10% T, and others)
Group 5	Innovators developing in the fields of activity A–S (40% C, 30% E, 20% B, 10% J, 10% D, and others)

## Results

Below are the PF identified in the market research and contribute to organizational involvement in sustainable development.

### Identifying Psychosocial Factors for Sustainable Development

Following in-depth interviews with the five groups of interviewees, the following data were obtained to identify PF that contribute to addressing sustainability in various activity fields. Each group was interviewed using the 10 general questions and the five personalized ones. Following these factors’ application, a series of PF were identified based on the framework analyses, following the analysis of the obtained data and the correlation with the framework analyses. The percent of the innovators obtained from the tabular situation of the types of innovators in the period 2015–2019 in Romania and the situation presented in Table 9 highlight the following factors: learning (L), perceived behavior control (CPB), attitude (A), belief (B), norms applicable to the field (NSAF), behavioral intent (BI), SN, the perceived power of the approach (PPA), BI, and SN.

**TABLE 9:** The situation of the identified psychosocial factors.

**Psychosocial factors identified**	**Dimensions of the identified psychosocial factors (PF)**	**Percentage of innovators (PI)**
Product innovators in the fields of activity A–S	Perception (P), motivation (M), learning (L), perceived behavior control (CPB), attitude (A), belief (B), and rules specifically applicable to the field (NSAF)	11.58 (PI1)
Process innovators only in the fields of activity A–S	Attitudes (A), behavioral intention (BI), subjective norms (SN), and perceived power of action (PPA)	14.30 (PI2)
Innovators only of business processes in the fields of activity A–S	motivation (M), learning (L), and belief (B)	30.51 (PI3)
Product and process innovators in the fields of activity A–S	Attitudes (A), behavioral intent (BI), subjective norms (SN), and norms especially applicable to the field (NSAF)	29.78 (PI4)
Product innovators only in the fields of activity A–S	Perceived power of approach (PPA), motivation (M), learning (L), subjective norms (SN), and conviction (B).	12.08 (PI5)

For innovative products in the fields of activity A–S, dimensions of the identified PF that contribute to the increase of involvement in organizational sustainability are perception, motivation, learning, CPB, attitude, belief, and rules specifically applicable to the field. Indeed, innovative products require certain aspects of marketing. Here also intervenes the individual’s behavior, which is well defined by the level factors. For process innovators only in activity A–S, the following factors were obtained: attitudes, BI, SN, and perceived power of action. Innovative processes require an adequate interpretation of SN, and PPA must have a certain intensity. A creative and new attitude defines process innovators. For innovators only of business processes in activity A–S, the dimensions identified are motivation, learning, and belief. Business processes are dynamic due to the constantly changing economic conditions. As a result, lifelong learning and motivation are essential elements. All this is corroborated with belief. For product and process innovators in activity A–S, the dimensions obtained are attitudes, behavioral intent, SN, and norms especially applicable to the field (NSAF). Process and product innovators are directed toward the business environment’s dynamics, adopt the new norms, and have adequate behavior for innovative principles. Product innovators are defined by a desire for lifelong learning and increased motivation. All these aspects are correlated with the dynamics of activity and the power of action.

### Importance Levels

The following rules agreed and developed by experts in the field were used to establish the importance of PF. The rules are presented in Table 10. The following assessment accepted in the Delphi rounds with experts was used to calculate importance. These levels are essential for defining the framework for improving organizational sustainability involvement’s intensity. It can be seen that five important levels were obtained, which are calculated according to the total score of psychosocial factors (TPS).

**TABLE 10:** Rules established by experts involved in the Delphi method.

**Level**	**Rules set by experts**
Level I	If TPS_*i*_ 50 then PS_*i*_ ∈ *Level*1
Level II	If TPS_*i*_ 30 and TPS_*i*_ < 50 then PS_*i*_ ∈ *Level*2
Level III	If TPS_*i*_ >20 and TPS_*i*_ < 30 then PS_*i*_ ∈ *Level*3
Level IV	If TPS_*i*_ 12 then PS_*i*_ ∈ *Level*4

$$T\,P\,S_{i}=\sum_{5}^{i=1}P\,I_{i}$$

where *I* 1…5.

## The Transdisciplinary Approach of Correlating the Levels of Importance With the Levels of Intensity

Based on the multilevel approach presented previously and agreed upon by the experts of the existing fields of activity in Romania, the following pyramidal representation can be developed. The levels of importance are presented in Table 11.

**TABLE 11:** Levels of the importance of the identified psychosocial factors.

**Level**	**Psychosocial factors associated with the levels set by experts**	**Significant levels of sustainability**
Level I	M, A, L, B, and BI	Complete involvement covering the three dimensions: economic, social, and environmental
Level II	SN	Above-average involvement in sustainability reporting
Level III	PPA	Partial involvement on certain dimensions
Level IV	P and CPB	Covering a dimension with an economic or environmental predominance

**FIGURE 2:**
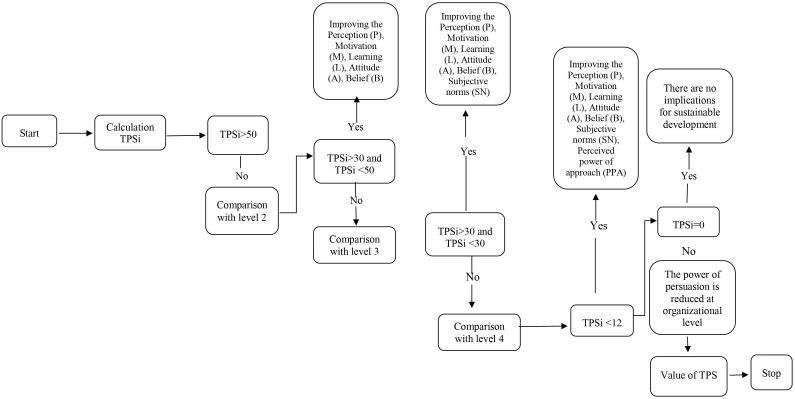
Simulating the improvement of psychosocial factors that contribute to organizational involvement in sustainable development.

The logical simulation of PF’ improvement is presented in Figure 2.

## Discussion and Conclusion

Following the research, an image is outlined on the PF that contribute to applying the principles of sustainability within the organization. The following discussions and future directions can be outlined. The research period was COVID-19, and the participants in the investigation stressed the importance of a strong motivation for adoption and involvement in further efforts. The COVID-19 period emphasized the need for a positive attitude to identifying new organizational opportunities. For the investigation, working groups were set up according to the organization’s level of innovation. The methods used in the present research, in-depth interviews, and the Delphi method were selected because they were considered effective methods for the research objectives. In-depth interviews provide concise, complex, and unambiguous results due to an expert who interviews each group.

Moreover, at the interviewed groups’ level, there were no deviations from the subject of tensions. These results were input for the Delphi method, with which experts in the field were able to generate a final report on PF involved in sustainable development. The Delphi method is a method that contributes to obtaining complex results in a short time with existing participants anywhere in the world.

In Romania, product and process innovations are numerous. Small and medium-sized organizations also have a high level of innovation. Simultaneously, the industry is involved in innovation, reaching a significant percentage. These working groups answered the 10 general questions, to which 5 personalized questions were added. The 20 experts outlined the PF that contribute to applying the principles of sustainability. Existing framework analyses were used. Following the identification, the four levels of importance were defined.

All experts have agreed on these levels. Level 1 includes perception (P), motivation (M), learning (L), attitude (A), belief (B), and BI. The second level comprises SN. The third level includes the PPA. The fourth level includes control of perceived behavior (CPB) and perception (P). Improving these factors contributes to improving organizational implications in SD. Stakeholders have an important role to play in applying innovative concepts. During the COVID-19 period, stakeholders showed some restraint, and certain negative attitudes were accentuated.

Correlating the level of importance of PF with the level of intensity of involvement in organizational sustainability contributes to identifying directions that need to be improved to increase the organization’s capacity to innovate and be involved in sustainability. This research covers certain gaps at the national level regarding a clear and objective identification of psychosocial factors.

The research’s future directions aimed at developing customized analysis frameworks for each field of activity. These frameworks will be correlated with important levels of sustainability.

## Data Availability Statement

The raw data supporting the conclusions of this article will be made available by the authors, without undue reservation.

## Author Contributions

All authors listed have made a substantial, direct and intellectual contribution to the work, and approved it for publication.

## Conflict of Interest

The authors declare that the research was conducted in the absence of any commercial or financial relationships that could be construed as a potential conflict of interest.

## Appendix 1

**TABLE 1:** Active enterprises, by activities of the national economy (National Institute of Statistics, 2020).

	**Year**						
	**2015**	**2016**	**2017**	**2018**	**2019**	**2020**	**2020**
	
	**Number**	**Number**	**Number**	**Number**	**Number**	**Number**	**Percent**
Total	554,967	507,440	513,850	527,792	553,796	576,545	100
Agriculture, forestry, and fishing (A)	13,602	17,471	18,396	19,139	19,916	20,514	3.56
Extractive industry (B)	1,083	1,112	1,107	1,076	1,014	1,033	0.18
Manufacturing industry (C)	57,305	48,090	48,404	48,347	49,837	52,451	9.10
Production and supply of electricity and heat, gas, hot water, and air conditioning (D)	506	1,503	1,460	1,350	1,206	1,200	0.21
Water distribution; sanitation, waste management, decontamination activities (E)	2,366	3,160	3,049	2,968	3,022	3,074	0.53
Construction (F)	59,389	47,814	48,341	49,716	52,792	55,978	9.71
Wholesale and retail trade; repair of motor vehicles and motorcycles (G)	214,137	176,202	171,959	169,712	172,435	172,856	29.98
Transport and storage (H)	34,489	39,666	41,746	44,504	48,382	51,944	9.01
Hotels and restaurants (I)	23,653	25,111	25,497	25,612	26,414	27,182	4.71
Information and communications (J)	20,049	19,499	20,619	22,012	23,837	25,452	4.41
Financial intermediation and insurance (K)	6,840	6,903	7,244	8,225	8,220	8,461	1.47
Real estate transactions (L)	14,767	13,844	14,472	15,349	167,04	17,867	3.10
Professional, scientific, and technical activities (M)	59,181	56,886	57,812	60,324	63,350	66,739	11.58
Administrative and support service activities (N)	19,480	19,406	19,965	20,802	22,285	22,848	3.96
Education (P)	2,681	3,772	4,252	4,942	5,811	6,393	1.11
Health and social work (Q)	8,677	10,093	10,959	13,188	15,251	17,114	2.97
Entertainment, cultural, and recreational activities (R)	4,990	5,758	6,778	7,740	9,003	9,945	1.72
Other service activities (S)	11,772	11,150	11,790	12,786	14,317	15,494	2.69
